# Health-related quality of life (HRQoL) of women with breast cancer undergoing treatment in a tertiary care centre in India

**DOI:** 10.1186/s41687-026-01071-8

**Published:** 2026-04-21

**Authors:** Abhilash Patra, Shona Nag, Shriniwas Subhash Kulkarni, Rebecca DeSouza, Hira B. Pant, Varun Agiwal, A. Y. Nirupama, G. V. S. Murthy

**Affiliations:** 1 Indian Institute of Public Health, Survey No: 384, Near Outer Ring Road Exist 17, Himayatsagar, Rajendra Nagar Mandal, Hyderabad, Telangana 500086 India; 2 Nag Foundation, Pune, Maharashtra India; 3 Oncology Department, Sahyadri Group of Hospital, Pune, Maharashtra India

**Keywords:** Breast cancer, Treatment, Quality of life (QoL), EORTC, India

## Abstract

**Introduction:**

Breast cancer in India accounts for 178361 of all cancers (Globocan 2020 report). Treatment causes financial, social, and psychological stress, impacting patients’ quality of life (QoL). Multiple studies have reported QoL among survivors, however few studies focused on patients undergoing treatment in India. Hence, the present study aimed to measure the QoL and its influencing factors associated with breast cancer undergoing various treatments in the oncology department of a tertiary care hospital in India.

**Methods:**

A hospital-based cross-sectional study (Nov 2022 - Jan 2023) used a semi-structured questionnaire on participants’ sociodemographic and clinical characteristics. The validated Marathi and English EORTC QLQ-C30 and BR23 assessed QoL. Participants were selected using convenience sampling.

**Results:**

A total of 285 patients agreed to participate with a mean age of 54.2 ± 12.2 years and the mean score of Global Health Status (GHS)/QoL was 54.4 ± 19.6. Patients who received radiation had more pain (*p* < 0.001), insomnia (*p* < 0.001), and diarrhoea (*p* = 0.005), whereas, those who received chemotherapy had higher scores in constipation (*p* < 0.001) and loss of appetite (*p* = 0.001). Co-morbidities were significantly associated with breast cancer-specific symptoms scales – upset by hair loss (*p* = 0.025), arm symptoms (*p* = 0.026), and breast symptoms (*p* = 0.013).

**Conclusion:**

Education, occupation, hours of sleep, financial issues, and type of surgery were linked to the patient’s QoL. Therefore, these patients require additional attention and support services, including effective pain management and interventions to enhance sleep quality, to further enhance their QoL. Longitudinal studies incorporating detailed clinical and psychosocial variables are needed to understand the determinants of QoL.

**Supplementary Information:**

The online version contains supplementary material available at 10.1186/s41687-026-01071-8.

## Abstract (plain language summary)

Despite the significant financial, social, and psychological stress associated with breast cancer treatment, evidence on the quality of life (QoL) of patients currently undergoing treatment are context specific. While most studies emphasize breast cancer survivors, very few have explored QoL during the active treatment phase in Indian settings. Understanding QoL and its influencing factors during treatment is crucial for developing effective support services and interventions to improve patient outcomes. This study investigates the impact of different treatments (radiation, chemotherapy, and hormonal therapy) on various aspects of QoL, including pain, insomnia, diarrhoea, constipation, and appetite loss. It also examines how sociodemographic and clinical characteristics affect problems caused by breast cancer or its treatment. The findings reveal that patients undergoing radiation therapy report higher levels of pain, insomnia, and diarrhoea, while those receiving chemotherapy experience more constipation and appetite loss. Additionally, patients who sleep less than eight hours have worse functional scales and report more pain, nausea/vomiting, and dyspnoea compared to those who sleep eight hours or more. These results highlight the distinct impacts of different breast cancer treatments on various aspects of patients’ QoL. Moreover, co-morbidities exacerbate specific breast cancer symptoms. Hence, the study highlights that when doctors and researchers assess patients’ outcomes and well-being during cancer treatment, they should consider both medical and social aspects to better understand their needs and improve their care. The study also underscores the need for comprehensive care that addresses these different dimensions of QoL.

**Supplementary Information:**

The online version contains supplementary material available at 10.1186/s41687-026-01071-8.

## Background

Globally, breast cancer is the most common cancer [1]. Studies reported that, globally, the burden of breast cancer will cross 2 million by 2030 [2]. In 2020, Globocan data reported breast cancer in India as the number one cancer and accounted for 178,361 of all cancers and 90,408 of all cancer deaths [3, 4]. As per the report from the National Cancer Registry, it is reported that there has been a significant increase in the incidence rate of breast cancer in all Population Based Cancer Registries (PBCRs) over the years since 2012, with particularly significant increase in trends seen in Mumbai, Chennai, Delhi and Bhopal registries [5–7].

Breast cancer causes significant stress and psychological impact [8]. It also causes body image problems because of surgical procedures like mastectomy, chemotherapy, radiotherapy, and hormonal therapy [8]. The required frequent hospital visits and long waiting lists also create stress [8]. Also, investigation, surgery, and therapy affect patients financially [9]. It also affects daily activities and work, adding stress and decreasing patients’ quality of life (QoL) [9]. Other factors that affect patients’ QoL include younger age, education, socioeconomic status, employment and other financial factors [10]. These factors are often more intense during the active treatment phase where the patient simultaneously phase symptoms associated with the disease, treatment side effects, psychosocial and financial stressors.

The decline in the QoL of patients is more pronounced in developing countries, primarily because majority of individuals with breast cancer are diagnosed at an advanced stage, resulting in less effective treatment [11]. A systematic review by Maharani et al. reported several additional factors influencing the QoL, such as limited financial resources, social stigma, psychological stress and anxiety, depression, and reduced access to supportive care services, all of which may further exacerbate the decline in QoL [12].

Additionally, health related quality of life (HRQoL) is commonly measured using descriptive health state classification systems such as EORTC QLQ-C30, which assess multiple functional and symptom domains. In some studies, these measures are also used to derive utility values for economic evaluation [13]. In India, the mean utility value of breast cancer patients during diagnoses was found to be 0.628 and there was a significant deterioration in health-related parameters in early and advanced breast carcinoma [13, 14]. A study conducted on HRQoL post breast cancer surgery, undergoing chemotherapy, reported high levels of pain, fatigue, and insomnia [15]. Multiple studies have reported the QoL among breast cancer survivors who are post-chemotherapy and surgery [10, 13, 16–20]. Evidence from systematic review study summarised HRQoL among patients undergoing active treatment from low-and-middle income countries in Asia and reported substantial physical, psychological and financial burden during this phase. However, there is a growing body of evidence in Asian countries, only three studies from India examined treatment specific impacts on QoL [21]. 

With advancements in breast cancer treatment that have a positive impact on survival rates and incidence, a thorough understanding of the QoL of patients during treatment, illness, and remission is crucial. Hence, the present study aims to measure the QoL and its influencing factors associated with breast cancer undergoing various treatments in the oncology department of a tertiary care hospital in Pune, Maharashtra.

### Objectives

1. To assess the quality of life (QoL) in breast cancer patients undergoing various treatment modalities.
2. To identify the factors associated with quality of life (QoL) in breast cancer patients.

## Methods

### Study design, duration and setting

A hospital-based cross-sectional study was conducted from November 2022 to January 2023 in the Oncology department of one of the tertiary care hospitals in Pune. The selected study period ensured feasible patient recruitment as there was consistent patient attendance at the oncology department.

### Study participants

All the breast cancer patients above the age of 18 years, who attended day treatment centres, inpatients, and outpatients who visited the tertiary care hospital in Pune were included in the study. Men with breast cancer were excluded, and women who were not willing to consent, unable to respond to the questionnaire, or critically ill were also excluded. Day treatment patients were those who visited for their chemotherapy, inpatients were those with breast cancer who were admitted to the wards, and outpatients who were under treatment and visited for follow-up during the study duration.

### Sample size

The sample size was determined using the formula for estimating population mean: n = (N*Z^2^*σ^2^/((N-1)*E^2^ + Z^2^* σ^2^)), where N represents the population size, Z is the Z-score corresponding to the desired confidence level (Z = 1.96 for 95% confidence interval), σ is the population standard deviation and E is the margin of error. Based on the mean QoL score and standard deviation reported by Pandey et al. (Mean = 90.6, SD = 18.4) and considering that approximately 300 breast cancer patients visited the hospital in the past three months (this figure was used as an estimate of patient flow for feasibility and sample size estimation, rather than a fixed sampling frame), the required sample size was 299 to estimate a mean with 95% confidence interval with a margin of error of 0.05 [19].

### Sampling technique

A consecutive sampling method (a non-random sampling method which includes all eligible patients who are available and meet the inclusion criteria during a specific time period) was used to select the participants for the study.

### Instruments and scoring

A semi-structured questionnaire was developed that includes sociodemographic and clinical characteristics of the participants such as age, marital status, education, occupation, comorbidities, duration of illness, type of treatment, subtypes, and information on surgery. The European Organization for Research and Treatment of Cancer Quality of Life Questionnaire (EORTC QLQ)-C30 and EORTC QLQ-BR23 were used, which are widely used instruments to assess the HRQoL of breast cancer patients and are considered to be a reliable and valid tool to measure the QoL in women with breast cancer [22, 23]. A validated Marathi and English version of the EORTC QLQ-C30 and EORTC QLQ-BR23 was used to assess the quality of life [22, 23].

The EORTC QLQ-C30 includes five functional scales, three symptom scales, a global health status (GHS)/Quality of life (QoL) scale, and six single-item scales. The functional scale includes physical (5 items), role (2 items), emotional (4 items), social (2 items), and cognitive functioning (2 items). Two items each for the GHS/QoL scale, nausea or vomiting, and pain. Fatigue had three items, and the rest of all six scales had one item each [22].

The EORTC QLQ-BR23 included four functional scales that include body image (4 items), sexual functioning (2 items), Sexual enjoyment (1 item), and future perspective (1 item). There were four symptom scales – systemic therapy side effects (7 items), breast symptoms (4 items), arm symptoms (3 items), and upset by hair loss (1 item).

All the scoring was done based on the EORTC scoring manual [24]. All the items were assigned a score value of 1–4 Likert scale, where 1 – “not at all”, 2 – “a little”, 3 – “quite a bit” and 4 – “very much”. GHS/QoL was assigned a score value of 1–7 where 1 was “very poor” and 7 was “excellent”. The scores of each item were averaged and termed as raw scores. A linear transformation method was used to standardize each raw score. The final score ranged from 0 to 100. A high score on the functional scale and GHS/QoL scale represented a healthy level of functioning, whereas a high score on the symptoms scale represented a high level of problems.

### Data collection process

A self-administered questionnaire was filled out by the participants. The sociodemographic characteristics were self-reported and the clinical characteristics of the patients were extracted from the electronic medical records (EMR) of the hospital by trained physicians. All the participants were given sufficient and brief information on the study and written informed consent was obtained.

### Data analysis

Data was entered in an Excel sheet. Cleaning, coding, and data analysis were carried out in STATA version 14 software.

Descriptive statistics included the participants sociodemographic and clinical characteristics such as age, marital status, education level, employment status, presence of concurrent illness, duration of illness, type of treatment, information on surgery, and comorbidities. Qualitative data was presented as frequencies and percentages, whereas mean and standard deviation were recorded for quantitative data. Independent t-test and one-way ANOVA were employed to determine the difference in the mean score of QoL measured by the functional and symptoms scales of EORTC QLQ-C30 and BR23 across sociodemographic and clinical characteristics.

Linear regression model was carried out to identify the factors associated with EORTC QLQ-C30 and BR23 scales. A significance level of 0.05 was employed with 95% confidence interval for statistical inference. Variables associated with EORTC QLQ-C30 and BR23 with a p-value below 0.05 were considered statistically significant and were included in the regression model.

## Results

### Sociodemographic and clinical characteristics of patients

Among a total number of 300 patients, 285 patients agreed to participate in the study with a response rate of 95%. The mean age of the patients was 54.2 ± 12.2 years and all the patients attended formal education with the majority of them completing secondary level (46.7%) of education. More than three-fifths of the patients were housewives (79.7%) and had two or more children (69.8%). Three-fourths of the patients had less than 8 h of sleep (75.4%). Most of the patients had a monthly household income of more than Rs.25,000/- (84.2%), however, 87% of the patients stated that their savings were affected due to cancer treatment. This income distribution closely corresponds with the estimated median household income for urban areas in Pune, Maharashtra, which ranges between ₹15,000 to ₹25,000 per month, based on data from the Socio-Economic and Caste Census [25]. Regarding clinical characteristics, 48.4% of patients were Estrogen Receptor positive (ER+)/ Progesterone Receptor positive (PR+)/Human Epidermal Growth Factor Receptor 2 negative (HER2neu-) a subtype of breast cancer. Other breast cancer subtypes HER2neu + and Triple Negative Breast Cancer (TNBC) were 99 (34.8%) and 48 (16.8%), respectively. Nearly, three-fifths of the patients had surgery, 201 (70.5%) had undergone chemotherapy and nearly 166 (57.6%) of the patients had no comorbidities. Among the patients those who reported co-morbidities (*n* = 122), hypertension was the most common condition (*n* = 54), followed by diabetes mellitus (*n* = 46), thyroid disorders (*n* = 20) and other comorbidities (*n* = 2) (Table 1).

**Table 1:** Sociodemographic and clinical characteristics of breast patients

Characteristics	Frequency (*n* = 285)	%
**Age (In years)**		
< 45 years	71	24.9
45–60 years	125	43.9
≥ 61 years	89	31.2
**Education**		
Primary	39	13.7
Secondary	133	46.7
Senior Secondary	72	25.3
Graduation and above	41	14.3
**Marital status**		
Single	10	3.5
Married	241	84.6
Divorced	18	6.3
Widowed	16	5.6
**Occupation**		
Government	9	3.2
Private	27	9.5
Self Employed	11	3.8
Housewives	227	79.7
Pensioner	11	3.8
**Sleep per day (in hours)**		
< 8 h	215	75.4
≥ 8 h	70	24.6
**Time since date of diagnosis**		
≤ 12 months	166	58.3
13–60 months	114	40
≥ 61 months	5	1.7
**Consumed traditional medicine**		
Yes	68	23.9
No	217	76.1
**Type of traditional medicine consumed (** ***n*** ** = 68)**		
Ayurvedic	23	33.8
Homeopathic	41	60.3
Others	4	5.9
**Subtypes**		
HER2neu+	99	34.8
ER+/PR+/HER2neu-	138	48.4
TNBC	48	16.8
**Treatment type**		
Endocrine Therapy	30	10.5
Chemotherapy	201	70.5
Radiation Therapy	54	19
**Surgery**		
Yes	169	59.3
No	116	40.7
**Type of surgery (** ***n*** ** = 169)**		
BCS	113	66.9
MRM	56	33.1
**Comorbidities**		
Yes	122	42.4
No	166	57.6
**Monthly household Income (in rupees)**		
≤ 25,000	45	15.8
> 25,000	240	84.2
**Savings affected**		
Yes	248	87
No	37	13
**Faced financial problem during treatment**		
Yes	58	20.4
No	227	79.6

### Descriptive statistics of EORTC QLQ- C30 and BR23

The mean score of Global Health Status (GHS)/QoL of the breast cancer patients was 54.4 ± 19.6. The functional scales of EORTC QLQ-C30 were reported as physical (76.9 ± 20.6), role (84.1 ± 23.9), emotional (60.7 ± 28.8), cognitive (76.9 ± 19.0), and social function (69.8 ± 27.6). The highest scores of symptom scales of EORTC QLQ C-30 were fatigue (50.5 ± 29.6), whereas the lowest symptoms score was diarrhoea (15.6 ± 27.7). The body image (69.5 ± 24.0) and sexual functioning (7.5 ± 15.3) recorded the highest and lowest scores in the functional scale of EORTC QLQ-BR23 respectively. Breast (15.2 ± 14.2) and arm (24.8 ± 22.8) symptoms scored the lowest whereas upset by hair loss (58.2 ± 35.5) scored the highest in the symptom’s scales and items (Table 2).

**Table 2:** Mean and standard deviation of EORTC QLQ C-30 and BR-23 components for breast cancer patients

EORTC QLQ C-30	Mean & SD	EORTC QLQ BR23	Mean & SD
**Global Health Status**	54.4 ± 19.6		
**Functional Scale**		**Symptoms Scales/items**	
Physical Function	76.9 ± 20.6	Systemic therapy Side Effects	35.2 ± 20.9
Role Function	84.1 ± 23.9	Upset by hair loss	58.2 ± 35.5
Emotional Function	60.7 ± 28.8	Arm Symptoms	24.8 ± 22.8
Cognitive Function	76.9 ± 19	Breast Symptoms	15.2 ± 14.2
Social Function	69.8 ± 27.6		
**Symptoms Scale**		**Functional Scale/items**	
Fatigue	50.5 ± 29.6	Body Image	69.5 ± 24.0
Nausea and vomiting	18.8 ± 26.7	Future Perspective	52.9 ± 35.9
Pain	37.7 ± 30.4	Sexual Functioning	7.5 ± 15.3
Dyspnoea	19.2 ± 23.4	Sexual Enjoyment	47.5 ± 30.1
Insomnia	47.4 ± 43.1		
Appetite loss	28.1 ± 32.9		
Constipation	19.6 ± 30.7		
Diarrhoea	15.6 ± 27.7		
Financial difficulties	39.5 ± 31.9		

### Association of EORTC QLQ-C30 and QLQ-BR23 scales with sociodemographic and clinical characteristics

#### Association of EORTC QLQ-C30

GHS/QoL (*p* = 0.001), physical (*p* = 0.029), role (*p* = 0.001), and emotional (*p* = 0.041) functioning had a significant association with education. Occupation was significantly associated with GHS/QoL (*p* = 0.023) and physical function (*p* = 0.036). Patients who had 8 h or more sleep per day had significantly better GHS/QoL (*p* = 0.001), physical (*p* = 0.008), role (*p* = 0.001), emotional (*p* = 0.001) and cognitive (*p* = 0.001) scores. Also, patients who underwent breast-conserving surgery (BCS) had better physical (*p* = 0.020), role (*p* = 0.003), emotional (*p* = 0.012) and social (*p* = 0.054) scores as compared to those underwent mastectomy (Table 3).

**Table 3:** EORTC-QLQ-C30 functional scores by demographic and clinical characteristics among breast cancer patients undergoing treatment in tertiary care hospital of Pune, Maharashtra

Characteristics	Global Health Status (GHS)/QoL	Physical Function	Role Function	Emotional Function	Cognitive Function	Social Function
**Age (In Years)**						
< 45 years	52.2 ± 18.6	76.2 ± 21.7	79.8 ± 24.9	60.2 ± 29.1	77.9 ± 18.6	60.3 ± 29.3
45–60 years	54.5 ± 20.3	77.6 ± 20.0	85.4 ± 23.7	63.0 ± 28.4	77.7 ± 19.2	72.8 ± 25.3
≥ 61 years	55.9 ± 19.2	76.4 ± 20.4	85.7 ± 23.2	57.7 ± 29.0	74.9 ± 18.9	73.4 ± 27.9
**p-value** ^**a**^	0.482	0.871	0.219	0.411	0.491	0.003*
**Education**						
Primary	58.1 ± 21.1	83.5 ± 15.6	94.0 ± 12.9	71.1 ± 20.8	78.2 ± 19.9	73.5 ± 26.9
Secondary	52.0 ± 19.3	75.0 ± 22.2	85.1 ± 23.7	59.4 ± 32.4	76.9 ± 16.2	72.8 ± 25.3
Senior Secondary	60.5 ± 17.5	79.7 ± 16.9	85.1 ± 22.3	61.5 ± 24.2	78.4 ± 22.2	63.8 ± 29.4
Graduation and above	47.7 ± 19.1	71.8 ± 22.9	69.5 ± 29.0	53.2 ± 28.2	72.7 ± 20.3	67.4 ± 31.1
**p-value** ^**a**^	0.001*	0.029*	0.001*	0.041*	0.456	0.119
**Marital Status**						
Single	64.9 ± 24.1	83.3 ± 21.8	90 ± 21.0	70.8 ± 25.5	83.3 ± 19.2	78.3 ± 30.4
Married	53.9 ± 18.9	75.9 ± 20.5	84.0 ± 24.0	59.4 ± 29.0	76.4 ± 18.5	69.4 ± 27.7
Divorced	53.7 ± 19.6	81.1 ± 24.2	75.9 ± 29.2	64.3 ± 28.4	79.6 ± 19.4	62.9 ± 27.7
Widowed	55.2 ± 25.6	83.3 ± 15.2	90.6 ± 14.8	68.7 ± 26.2	77.0 ± 25	79.1 ± 22.3
**p-value** ^**a**^	0.380	0.285	0.273	0.361	0.645	0.270
**Occupation**						
Government	63.8 ± 16.1	82.2 ± 9.4	81.4 ± 29.3	75.9 ± 10.5	85.1 ± 19.4	72.2 ± 16.6
Private	62.3 ± 18.2	87.1 ± 15.3	89.5 ± 21.7	60.4 ± 32.4	80.8 ± 19.4	64.8 ± 30.4
Self Employed	56.8 ± 24.9	83.0 ± 23.1	81.8 ± 24.0	59.8 ± 26.5	83.3 ± 22.3	69.6 ± 22.1
Housewives	52.5 ± 19.2	75.1 ± 21.0	83.3 ± 24.1	59.4 ± 28.9	75.4 ± 18.5	70.2 ± 27.9
Pensioner	63.6 ± 19.1	79.3 ± 18.0	92.4 ± 20.2	75 ± 24.1	84.8 ± 21.6	72.7 ± 30.0
**p-value** ^**a**^	0.023*	0.036*	0.534	0.227	0.112	0.891
**Sleep per day (in hours)**						
< 8 h	51.6 ± 19.6	75.1 ± 22.0	81.4 ± 25.5	57.5 ± 29.0	74.6 ± 19.0	68.9 ± 28.6
≥ 8 h	62.9 ± 16.8	82.5 ± 14.0	92.3 ± 15.7	70.2 ± 26.1	83.8 ± 17.2	72.6 ± 24.2
**p-value** ^**b**^	0.001*	0.008*	0.001*	0.001*	0.001*	0.341
**Time since date of diagnosis**						
≤ 12 months	56.3 ± 18.1	79.9 ± 18.0	86.0 ± 23.3	59.6 ± 27.5	77.8 ± 19.1	69.4 ± 27.9
13–60 months	51.8 ± 21.4	73.2 ± 23.0	81.6 ± 24.2	62.1 ± 30.7	75.5 ± 18.8	71.0 ± 26.5
≥ 61 months	50 ± 15.5	62.6 ± 23.3	76.6 ± 32.4	63.3 ± 29.2	76.6 ± 22.3	56.6 ± 45.0
**p-value** ^**a**^	0.147	0.008*	0.256	0.760	0.630	0.503
**Consumed traditional medicine**						
Yes	56.0 ± 19.1	72.9 ± 20.5	81.1 ± 24.4	60.0 ± 29.3	79.6 ± 19.9	67.8 ± 31.0
No	53.9 ± 19.7	78.2 ± 20.4	85.0 ± 23.7	60.9 ± 28.7	76.0 ± 18.6	70.5 ± 26.5
**p-value** ^**b**^	0.444	0.064	0.234	0.830	0.171	0.497
**Savings Affected**						
Yes	53.6 ± 19.5	75.9 ± 20.7	83.9 ± 24.0	58.8 ± 29.1	75.5 ± 18.7	69.6 ± 27.2
No	59.6 ± 19.3	83.9 ± 17.9	85.1 ± 23.1	73.1 ± 23.2	86.0 ± 18.2	71.1 ± 30.5
**p-value** ^**b**^	0.079	0.026*	0.786	0.004*	0.001*	0.762
**Faced Financial problems**						
Yes	48.5 ± 17.8	70 ± 23.1	79.0 ± 27.3	50 ± 29.2	70.9 ± 16.6	66.6 ± 26.4
No	55.9 ± 19.7	78.7 ± 19.5	85.5 ± 22.8	63.4 ± 28.1	78.4 ± 19.3	70.7 ± 27.9
**p-value** ^**b**^	0.010*	0.003*	0.067	0.001*	0.007*	0.322
**Subtypes**						
HER2neu+	57.4 ± 22.2	77.1 ± 21.3	84.2 ± 24.5	62.6 ± 26.6	78.7 ± 20.0	68.5 ± 28.9
ER+/PR+/HER2neu-	52.9 ± 17.2	79.1 ± 19.3	86.0 ± 22.2	60.3 ± 31.2	76.5 ± 16.1	73.1 ± 26.9
TNBC	52.4 ± 19.6	70.2 ± 21.4	78.4 ± 26.8	57.8 ± 26.0	73.9 ± 23.7	63.1 ± 25.9
**p-value** ^**a**^	0.168	0.035*	0.170	0.620	0.339	0.081
**Treatment type**						
Endocrine Therapy	56.0 ± 23.7	73.6 ± 25.8	84.5 ± 27.5	59.7 ± 32.0	75 ± 18.9	77.2 ± 27.1
Chemotherapy	54.6 ± 19.6	77.2 ± 20.5	81.3 ± 24.8	64.6 ± 26.1	78.4 ± 20.4	65.5 ± 27.7
Radiation Therapy	52.7 ± 16.6	77.8 ± 17.5	94.1 ± 13.6	46.4 ± 32.0	72.2 ± 11.2	81.7 ± 23.1
**p-value** ^**a**^	0.737	0.639	0.002*	< 0.001*	0.086	< 0.001*
**Surgery**						
Yes	58.7 ± 18.0	79.1 ± 17.8	88.3 ± 21.1	60.5 ± 29.4	77.2 ± 18.1	76.6 ± 21.9
No	48.1 ± 20.0	73.7 ± 23.6	77.9 ± 26.3	60.9 ± 27.9	76.4 ± 20.2	60.0 ± 31.9
**p-value** ^**b**^	< 0.001*	0.028*	< 0.001*	0.916	0.733	< 0.001*
**Type of Surgery (** ***n*** ** = 169)**						
BCS	59.3 ± 17.6	81.3 ± 16.2	91.7 ± 18.2	68.6 ± 26.8	78.3 ± 14.0	78.9 ± 23.1
MRM	57.4 ± 19.0	74.7 ± 20.2	81.5 ± 24.9	56.5 ± 29.9	75 ± 24.4	72.0 ± 18.8
**p-value** ^**b**^	0.514	0.020*	0.003*	0.012*	0.264	0.054*
**Comorbidities**						
Yes	55.5 ± 21.1	74.7 ± 21.1	83.9 ± 24.5	63.4 ± 27.3	76.9 ± 20.8	66.1 ± 29.7
No	53.5 ± 18.3	78.6 ± 20.0	84.2 ± 23.4	58.6 ± 29.7	76.8 ± 17.5	72.6 ± 25.7
**p-value** ^**b**^	0.378	0.113	0.913	0.163	0.992	0.045*
**Monthly household Income (in Rupees)**						
≤ 25,000	54.9 ± 22.5	80.2 ± 17.9	90.7 ± 20.5	65.7 ± 24.5	75.1 ± 19.3	73.7 ± 24.7
> 25,000	54.3 ± 19.0	76.3 ± 21.0	82.9 ± 24.3	59.7 ± 29.4	77.2 ± 18.9	69.1 ± 28.1
**p-value** ^**b**^	0.829	0.236	0.043*	0.201	0.51	0.313

Note: ^a^One Way Anova; ^b^ T-test; *significant at 5% level of significance

Age was significantly associated with EORTC QLQ-C30 symptoms scale – diarrhoea (*p* = 0.029) and financial difficulties (*p* = 0.005). Patients who received radiation had more pain (*p* < 0.001), insomnia (*p* = < 0.001) and diarrhoea (*p* = 0.005), whereas, those who received chemotherapy had significantly higher scores in constipation (*p* < 0.001) and loss of appetite (*p* = 0.001). Patients who slept less than 8 h per day had more fatigue, nausea and vomiting, pain, dyspnoea and insomnia as compared to those who slept more than 8 h per day and were significantly associated (Table 4).

**Table 4:** EORTC-QLQ-C30 symptoms scores by demographic and clinical characteristics among breast cancer patients undergoing treatment in tertiary care hospital of Pune, Maharashtra

Characteristics	Fatigue	Nausea and vomiting	Pain	Dyspnoea	Insomnia	Appetite loss	Constipation	Diarrhoea	Financial difficulties
**Age (In Years)**									
< 45 years	47.4 ± 31.0	21.0 ± 24.8	33.5 ± 30.2	19.4 ± 23.5	43.6 ± 42.3	28.6 ± 34.8	20.1 ± 30.5	16.4 ± 28.1	49.7 ± 35.5
45–60 years	50.2 ± 30.1	18.1 ± 28.9	36.5 ± 29.9	19.8 ± 25.2	43.7 ± 43.0	28.2 ± 33.3	18.9 ± 29.4	11.2 ± 25.7	37.6 ± 29.0
≥ 61 years	53.4 ± 27.6	17.9 ± 25.0	42.8 ± 30.9	18.3 ± 20.7	55.4 ± 43.2	27.3 ± 31.2	20.2 ± 32.8	21.3 ± 29.4	34.0 ± 31.3
**p-value** ^**a**^	0.432	0.718	0.130	0.895	0.104	0.966	0.941	0.029*	0.005*
**Education**									
Primary	49.2 ± 26.8	7.2 ± 13.1	32.0 ± 24.5	17.0 ± 27.4	46.1 ± 35.5	29.9 ± 36.5	24.7 ± 30.3	5.1 ± 14.3	32.4 ± 25.9
Secondary	52.2 ± 31.1	20.7 ± 28.3	42.6 ± 33.4	20.0 ± 22.6	55.1 ± 45.1	25.8 ± 31.6	14.5 ± 27.6	16.2 ± 28.0	36.5 ± 32.0
Senior Secondary	48.3 ± 28.1	18.2 ± 27.2	29.3 ± 26.0	17.1 ± 23.7	35.1 ± 40.2	26.3 ± 32.5	30.0 ± 35.8	14.3 ± 28.9	43.5 ± 30.4
Graduation and above	50.1 ± 30.0	24.3 ± 27.6	42.2 ± 29.1	22.7 ± 21.6	44.7 ± 43.8	36.5 ± 33.9	13.0 ± 25.6	26.0 ± 31.1	48.7 ± 37.3
**p-value** ^**a**^	0.823	0.019*	0.01*	0.575	0.015*	0.300	0.001*	0.008*	0.054*
**Marital status**									
Single	32.2 ± 29.8	11.6 ± 17.6	26.6 ± 28.5	6.6 ± 14.0	16.6 ± 36.0	20 ± 28.1	6.6 ± 21.0	6.6 ± 21.0	43.3 ± 35.3
Married	51.2 ± 29.0	19.7 ± 27.6	39.3 ± 30.4	19.2 ± 23.3	48.5 ± 43.4	28.4 ± 32.7	20.4 ± 31.1	15.9 ± 28.0	39.8 ± 31.6
Divorced	43.2 ± 33.8	13.8 ± 19.1	23.1 ± 25.6	22.2 ± 22.2	42.5 ± 42.4	22.2 ± 32.3	14.8 ± 28.5	18.5 ± 30.7	46.2 ± 39.8
Widowed	59.0 ± 29.1	14.5 ± 24.2	37.5 ± 32.4	25.0 ± 28.5	54.1 ± 38.2	33.3 ± 40.3	20.8 ± 31.9	14.5 ± 24.2	25 ± 22.7
**p-value** ^**a**^	0.092	0.569	0.104	0.248	0.117	0.658	0.481	0.735	0.233
**Occupation**									
Government	40.7 ± 34.6	7.4 ± 12.1	11.1 ± 18.6	14.8 ± 24.2	11.1 ± 33.3	0	3.7 ± 11.1	3.7 ± 11.1	33.3 ± 16.6
Private	40.7 ± 34.0	16.6 ± 29.9	32.0 ± 27.7	7.4 ± 16.8	34.5 ± 40.8	7.4 ± 19.2	4.9 ± 15.2	11.1 ± 22.6	49.3 ± 33.8
Self Employed	38.3 ± 36.2	10.6 ± 15.4	25.7 ± 32.7	6.0 ± 13.4	24.2 ± 33.6	30.3 ± 34.8	21.2 ± 34.2	24.2 ± 39.6	36.3 ± 37.8
Housewives	52.9 ± 28.6	20.3 ± 27.3	41.0 ± 30.2	22.0 ± 24.0	52.5 ± 42.7	31.7 ± 33.6	22.0 ± 32.0	16.7 ± 28.2	39.0 ± 31.5
Pensioner	45.4 ± 19.5	10.6 ± 20.1	18.1 ± 28.3	9.0 ± 15.5	24.2 ± 42.4	24.2 ± 30.1	18.1 ± 27.3	6.0 ± 20.1	33.3 ± 39.4
**p-value** ^**a**^	0.106	0.332	0.001*	0.002*	< 0.001*	< 0.001*	0.038*	0.277	0.484
**Sleep per day (in hours)**									
< 8 h	52.6 ± 29.8	20.7 ± 27.9	41.6 ± 29.5	21.7 ± 23.8	53.9 ± 42.0	29.7 ± 33.0	20.9 ± 31.2	16.2 ± 28.0	41.5 ± 32.0
≥ 8 h	44.1 ± 27.9	12.8 ± 21.8	25.9 ± 30.2	11.9 ± 20.4	27.1 ± 40.2	22.8 ± 32.3	15.7 ± 28.7	13.8 ± 26.9	33.3 ± 31.0
**p-value** ^**a**^	0.036*	0.031*	< 0.001*	0.002*	< 0.001*	0.128	0.217	0.519	0.061
**Time since date of diagnosis**									
≤ 12 months	51.6 ± 29.6	15.8 ± 25.0	37.9 ± 31.6	17.0 ± 23.4	49.7 ± 42.7	25.1 ± 32.9	20.2 ± 31.9	15.4 ± 27.8	38.5 ± 32.8
13–60 months	48.3 ± 30.0	23.3 ± 28.7	37.4 ± 28.7	22.2 ± 23.4	42.9 ± 43.6	31.2 ± 32.9	17.5 ± 27.7	16.0 ± 27.7	40.3 ± 30.5
≥ 61 months	62.2 ± 9.9	13.3 ± 21.7	40 ± 25.2	26.6 ± 14.9	66.6 ± 40.8	53.3 ± 18.2	46.6 ± 44.7	13.3 ± 29.8	53.3 ± 38.0
**p-value** ^**a**^	0.439	0.063	0.976	0.152	0.259	0.067	0.106	0.965	0.560
**Consumed traditional medicine**									
Yes	58.4 ± 28.5	18.1 ± 25.2	38.4 ± 26.1	21.0 ± 22.9	50 ± 44.2	28.9 ± 32.0	25.4 ± 35.0	9.8 ± 23.0	42.1 ± 27.3
No	48.0 ± 29.5	19.0 ± 27.2	37.5 ± 31.7	18.7 ± 23.6	46.5 ± 42.7	27.8 ± 33.3	17.8 ± 29.0	17.5 ± 28.8	38.7 ± 33.2
**p-value** ^**b**^	**0.01**	0.812	0.827	0.474	0.565	0.807	0.072	0.045*	0.437*
**Savings Affected**									
Yes	51.9 ± 29.7	19.9 ± 27.3	39.0 ± 30.4	19.6 ± 23.1	48.2 ± 42.7	28.6 ± 32.8	18.5 ± 29.1	16.1 ± 28.2	41.8 ± 31.8
No	41.1 ± 26.8	11.2 ± 20.8	29.3 ± 29.2	17.1 ± 25.6	41.4 ± 46.0	24.3 ± 33.9	27.0 ± 34.9	12.6 ± 24.0	24.3 ± 29.0
**p-value** ^**b**^	**0.038**	0.065	0.068	0.545	0.371	0.459	0.117	0.473	0.001*
**Faced financial problems**									
Yes	58.0 ± 30.1	22.9 ± 26.4	45.1 ± 30.7	26.4 ± 22.3	58.6 ± 42.0	28.1 ± 32.3	15.5 ± 29.4	22.4 ± 31.4	50.5 ± 33.7
No	48.6 ± 29.1	17.7 ± 26.7	35.9 ± 30.1	17.4 ± 23.4	44.4 ± 43.0	28.0 ± 33.2	20.7 ± 30.9	13.9 ± 26.5	36.7 ± 30.9
**p-value** ^**b**^	**0.029**	0.183	0.183	0.009*	0.025*	0.981	0.251	0.038*	0.003*
**Subtypes**									
HER2neu+	50.7 ± 25.8	15.6 ± 25.4	32.4 ± 26.5	18.3 ± 23.2	34.3 ± 41.6	29.9 ± 32.4	21.2 ± 29.5	12.7 ± 27.2	46.1 ± 33.2
ER+/PR+/HER2neu-	48.6 ± 32.6	20.0 ± 27.9	41.4 ± 32.8	18.9 ± 21.2	55.3 ± 41.9	22.9 ± 31.4	16.9 ± 30.2	19.8 ± 29.2	35.7 ± 31.1
TNBC	55.5 ± 27.2	21.8 ± 25.5	38.1 ± 29.7	22.2 ± 29.4	51.3 ± 44.0	38.8 ± 35.9	24.3 ± 34.2	9.7 ± 22.7	36.8 ± 30.1
**p-value** ^**a**^	0.376	0.32	0.082	0.627	< 0.001*	0.011*	0.293	0.041*	0.032*
**Treatment type**									
Endocrine Therapy	42.9 ± 30.5	19.8 ± 31.3	31.6 ± 27.4	20.5 ± 22.1	36.6 ± 40.4	27.7 ± 30.4	13.3 ± 31.0	12.2 ± 25.4	46.6 ± 34.5
Chemotherapy	51.0 ± 28.2	17.8 ± 24.6	32.7 ± 26.7	19.9 ± 25.1	41.7 ± 41.6	32.0 ± 33.9	24.3 ± 32.1	13.2 ± 25.6	41.1 ± 30.9
Radiation Therapy	52.6 ± 33.4	21.8 ± 31.3	59.8 ± 35.0	16.3 ± 16.6	74.0 ± 40.2	13.5 ± 26.3	5.5 ± 18.0	26.5 ± 33.8	29.6 ± 32.8
**p-value** ^**a**^	0.315	0.614	< 0.001*	0.587	< 0.001*	0.001*	< 0.001*	0.005*	0.027*
**Surgery**									
Yes	49.3 ± 31.0	15.3 ± 25.9	40.6 ± 31.0	17.3 ± 23.8	48.3 ± 13.4	22.8 ± 30.9	18.3 ± 30.1	16.1 ± 28.4	35.5 ± 30.4
No	52.2 ± 27.2	23.8 ± 27.1	33.6 ± 29.2	22.1 ± 22.6	45.9 ± 42.7	35.6 ± 34.5	21.5 ± 31.4	14.9 ± 26.8	45.4 ± 33.3
**p-value** ^**b**^	0.402	0.008*	0.054*	0.0916	0.652	0.001*	0.387	0.713	0.010*
**Type of Surgery (** ***n*** ** = 169)**									
BCS	47.2 ± 31.7	16.5 ± 27.4	43.0 ± 33.5	18.5 ± 22.6	52.2 ± 45.3	19.4 ± 29.1	14.4 ± 26.3	17.6 ± 31.2	36.5 ± 31.7
MRM	53.3 ± 29.6	13.0 ± 22.8	35.7 ± 24.7	14.8 ± 26.1	40.4 ± 38.5	29.7 ± 33.4	26.1 ± 35.7	13.0 ± 21.7	33.3 ± 27.7
**p-value** ^**b**^	0.232	0.421	0.147	0.344	0.098	0.041*	0.016*	0.323	0.516
**Comorbidities**									
Yes	54.0 ± 26.6	17.4 ± 24.2	40.7 ± 32.5	17.6 ± 21.7	40.9 ± 41.5	28.1 ± 30.6	21.8 ± 32.0	12.5 ± 23.6	38.7 ± 30.7
No	47.8 ± 31.4	19.8 ± 28.4	33.7 ± 26.9	20.5 ± 24.6	52.1 ± 43.8	28.0 ± 34.7	17.9 ± 29.6	17.9 ± 30.3	40.0 ± 32.9
**p-value** ^**b**^	0.077	0.470	0.052*	0.297	0.030*	0.974	0.294	0.102	0.737
**Monthly household Income (in Rupees)**									
≤ 25,000	47.4 ± 29.1	14.8 ± 20.7	32.5 ± 26.5	11.1 ± 15.8	37.7 ± 35.2	21.4 ± 25.7	24.4 ± 30.4	5.9 ± 16.3	46.6 ± 29.6
> 25,000	51.1 ± 29.6	19.5 ± 27.6	38.7 ± 31.0	20.8 ± 24.3	49.1 ± 44.2	29.3 ± 34.0	18.7 ± 30.7	17.5 ± 29.0	38.1 ± 32.2
**p-value** ^**b**^	0.441	0.275	0.213	0.010*	0.104	0.144	0.254	0.010*	0.102

Note: ^a^One Way Anova; ^b^ T-test; *significant at 5% level of significance

#### Association of EORTC QLQ-BR23

Older patients experienced fewer systemic therapy side-effects (*p* = 0.008) and breast symptoms (*p* = 0.044). Those with only primary education were more upset by hair loss compared to those with secondary education or above (*p* = 0.018). TNBC patients had significantly higher scores for systemic therapy side effects (*p* = 0.002) and breast symptoms (*p* = 0.053) compared to other subtypes. Subtypes were also significantly associated with lower sexual functioning (*p* = 0.038). Patients who underwent chemotherapy reported significantly higher systemic therapy side effects (*p* < 0.001) and were upset by hair loss symptoms scale scores (*p* = 0.030) as compared to endocrine and radiation therapy. Whereas those patients who underwent radiation therapy (*p* < 0.001) had significantly higher arm symptoms as compared those receiving chemotherapy or endocrine therapy.

Additionally, patients who underwent surgery reported higher arm symptom scores (*p* < 0.001) compared to those who did not. There was a significant association between co-morbidities and breast cancer-specific symptoms scales – upset by hair loss (*p* = 0.025), arm symptoms (*p* = 0.026), and breast symptoms (*p* = 0.013) (Table 5).

**Table 5:** EORTC-QLQ-BR23 functional and symptoms scores by demographic and clinical characteristics among breast cancer patients undergoing treatment in tertiary care hospital of Pune, Maharashtra

Characteristics	Systemic therapy Side Effects	Upset by hair loss	Arm Symptoms	Breast Symptoms	Body Image	Future Perspective	Sexual Functioning	Sexual Enjoyment
**Age (In Years)**								
< 45 years	40.4 ± 21.7	66.1 ± 33.8	21.2 ± 21.8	16.0 ± 14.1	68.3 ± 23.9	50.7 ± 38.5	9.2 ± 17.0	48.8 ± 24.7
45–60 years	35.6 ± 20.3	56.2 ± 37.0	24.5 ± 22.0	16.9 ± 15.9	67.8 ± 22.9	53.3 ± 34.6	8.1 ± 17.4	50.0 ± 38.9
≥ 61 years	30.2 ± 20.2	53.9 ± 33.9	28.2 ± 24.3	12.1 ± 10.8	72.8 ± 25.7	53.9 ± 35.7	5.2 ± 9.6	44.4 ± 29.5
**p-value** ^**a**^	0.008*	0.110	0.157	0.044*	0.285	0.837	0.228	0.890
**Education**								
Primary	42.0 ± 24.4	73.3 ± 25.3	17.0 ± 19.0	13.4 ± 11.5	72.2 ± 22.6	56.4 ± 30.7	7.6 ± 16.1	33.3
Secondary	30.1 ± 20.2	54.7 ± 35.0	29.5 ± 25.2	16.2 ± 16.3	70.7 ± 24.7	52.6 ± 37.4	7.1 ± 14.2	45.0 ± 37.1
Senior Secondary	41.1 ± 20.3	59.9 ± 41.4	23.1 ± 20.2	15.5 ± 12.1	69.0 ± 23.1	59.7 ± 34.0	7.8 ± 16.3	56.4 ± 25.0
Graduation and above	34.2 ± 17.1	48.4 ± 27.7	20.0 ± 19.1	13.0 ± 11.9	63.6 ± 24.7	38.2 ± 35.4	7.7 ± 16.7	46.6 ± 29.8
**p-value** ^**a**^	< 0.001*	0.018*	0.005*	0.508	0.346	0.019*	0.988	0.570
**Marital Status**								
Single	25.7 ± 20.2	55.5 ± 37.2	15.5 ± 18.2	13.3 ± 10.5	74.1 ± 24.6	76.6 ± 35.3	13.3 ± 23.3	33.3
Married	35.2 ± 20.4	59.3 ± 35.5	26.0 ± 23.2	16.2 ± 14.5	68.9 ± 24.2	52.0 ± 35.9	7.3 ± 14.9	49.0 ± 32.0
Divorced	37.0 ± 19.5	47.0 ± 37.3	16.6 ± 17.1	9.2 ± 11.0	68.5 ± 21.8	46.2 ± 36.4	10.2 ± 19.9	33.3
Widowed	38.0 ± 29.1	57.1 ± 33.1	21.5 ± 22.3	7.8 ± 8.8	76.5 ± 23.6	58.3 ± 31.0	3.1 ± 6.7	50 ± 23.6
**p-value** ^**a**^	0.477	0.586	0.169	0.028*	0.590	0.127	0.345	0.872
**Occupation**								
Government	35.4 ± 22.6	50 ± 53.4	11.1 ± 22.2	3.7 ± 4.3	70.3 ± 30.9	77.7 ± 23.5	1.8 ± 5.5	66.7
Private	26.2 ± 17.9	49.2 ± 42.9	30.4 ± 21.4	23.4 ± 14.6	73.7 ± 23.9	55.5 ± 36.9	14.2 ± 19.9	55.5 ± 34.4
Self Employed	36.3 ± 13.8	60.6 ± 29.1	24.2 ± 16.3	14.3 ± 17.1	62.1 ± 13.1	54.5 ± 37.3	9.0 ± 21.5	0
Housewives	36.3 ± 21.5	59.0 ± 34.2	25.2 ± 23.3	15.1 ± 13.9	68.6 ± 24.2	50.9 ± 35.6	7.0 ± 14.6	46.2 ± 30.6
Pensioner	29.8 ± 16.2	66.6 ± 33.3	16.1 ± 15.2	6.0 ± 8.4	83.3 ± 20.0	63.6 ± 40.7	3.0 ± 10.0	33.3
**p-value** ^**a**^	0.171	0.659	0.161	< 0.001*	0.214	0.188	0.105	0.794
**Sleep per day (in hours)**								
< 8 h	37.7 ± 20.8	59.0 ± 33.8	24.4 ± 22.7	15.8 ± 14.4	67.0 ± 24.4	48.6 ± 35.2	7.9 ± 16.0	45.1 ± 30.4
≥ 8 h	29.2 ± 20.1	55.9 ± 39.9	26.1 ± 23.2	13.3 ± 13.1	77.0 ± 21.3	65.7 ± 34.9	6.1 ± 13.0	58.3 ± 29.5
**p-value** ^**b**^	0.006*	0.556	0.579	0.196	0.002*	0.001*	0.417	0.280
**Time since date of diagnosis**								
≤ 12 months	35.6 ± 21.3	61.8 ± 34.6	23.1 ± 23.5	14.2 ± 12.5	71.2 ± 24.1	52.8 ± 35.6	4.2 ± 10.8	49.9 ± 23.5
13–60 months	33.7 ± 20.2	51.8 ± 35.7	27.7 ± 21.8	16.7 ± 16.3	66.6 ± 23.7	52.9 ± 36.5	12.2 ± 19.3	46.0 ± 35.7
≥ 61 months	48.5 ± 19.1	73.3 ± 43.4	15.5 ± 9.93	13.3 ± 9.5	76.6 ± 25.9	53.3 ± 38.0	6.6 ± 14.9	0
**p-value** ^**a**^	0.267	0.073	0.164	0.339	0.237	0.999	< 0.001*	0.690
**Consumed traditional medicine**								
Yes	39.4 ± 19.1	61.4 ± 39.0	24.0 ± 21.0	16.1 ± 13.2	71.2 ± 23.4	48.5 ± 37.0	7.3 ± 15.0	48.1 ± 24.2
No	33.7 ± 21.3	57.2 ± 34.4	25.1 ± 23.3	14.9 ± 14.4	68.9 ± 24.3	54.2 ± 35.4	7.5 ± 15.4	47.7 ± 32.3
**p-value** ^**b**^	0.048*	0.460	0.724	0.530	0.506	0.254	0.935	0.975
**Savings Affected**								
Yes	34.8 ± 20.3	59.1 ± 35.1	25.5 ± 23.2	15.6 ± 14.4	68.9 ± 24.1	52.0 ± 36.1	7.9 ± 16.0	44.1 ± 29.2
No	37.0 ± 24.5	52.0 ± 37.8	20.1 ± 19.5	12.3 ± 11.2	73.1 ± 23.4	58.5 ± 33.7	4.0 ± 8.2	73.3 ± 27.8
**p-value** ^**b**^	0.552	0.295	0.174	0.190	0.317	0.301	0.144	0.043*
**Faced Financial problems**								
Yes	35.0 ± 17.4	54.1 ± 30.1	23.9 ± 23.2	15.9 ± 11.8	64.3 ± 24.9	43.6 ± 38.0	8.9 ± 17.7	57.1 ± 25.2
No	35.1 ± 21.7	59.1 ± 36.7	25.1 ± 22.7	15.0 ± 14.7	70.8 ± 23.7	55.2 ± 35.0	7.1 ± 14.6	45.8 ± 31.4
**p-value** ^**b**^	0.968	0.413	0.729	0.667	0.068	0.028*	0.429	0.379
**Subtypes**								
HER2neu+	36.5 ± 17.4	63.2 ± 38.6	18.9 ± 19.5	15.9 ± 15.4	68.1 ± 23.9	53.8 ± 33.9	4.7 ± 11.4	43.1 ± 22.8
ER+/PR+/HER2neu-	31.4 ± 22.8	56.3 ± 33.3	29.7 ± 25.2	13.4 ± 12.6	72.9 ± 23.9	53.8 ± 37.2	9.7 ± 17.5	58.8 ± 34.4
TNBC	43.0 ± 19.4	61.6 ± 32.8	22.9 ± 18.3	18.9 ± 15.0	62.3 ± 23.4	47.9 ± 36.2	6.5 ± 14.8	26.6 ± 27.8
**p-value** ^**a**^	0.002*	0.175	0.001*	0.053*	0.024*	0.579	0.038*	0.076
**Treatment type**								
Endocrine Therapy	27.4 ± 16.8	42.4 ± 40.0	20 ± 18.0	15.5 ± 16.9	75 ± 21.1	50 ± 37.9	10.5 ± 17.2	66.6 ± 36.5
Chemotherapy	39.6 ± 19.6	61.2 ± 35.2	20.7 ± 19.2	16.1 ± 14.1	66.2 ± 23.4	54.2 ± 35.5	7.1 ± 16.2	43 ± 27.4
Radiation Therapy	22.6 ± 21.6	51.1 ± 29.9	42.7 ± 28.3	11.7 ± 12.2	78.5 ± 25.3	49.3 ± 36.4	7.1 ± 9.4	66.6 ± 47.1
**p-value** ^**a**^	< 0.001*	0.030*	< 0.001*	0.127	0.001*	0.611	0.512	0.146
**Surgery**								
Yes	31.9 ± 20.7	54.1 ± 37.0	31.6 ± 24.0	15.2 ± 12.9	71.7 ± 23.1	56.0 ± 35.5	7.4 ± 14.9	44.4 ± 25.5
No	39.8 ± 20.4	63.3 ± 32.8	14.9 ± 16.5	15.1 ± 15.8	66.2 ± 25.1	48.2 ± 36.0	7.4 ± 15.9	50.7 ± 34.3
**p-value** ^**b**^	0.001*	0.050*	< 0.001*	0.940	0.054*	0.073	0.989	0.522
**Type of Surgery (** ***n*** ** = 169)**								
BCS	28.0 ± 20.1	50.2 ± 36.9	28.7 ± 20.0	14.6 ± 12.0	68.6 ± 20.4	53.9 ± 37.0	6.5 ± 15.7	51.5 ± 27.3
MRM	39.7 ± 19.6	59.8 ± 36.8	33.1 ± 25.7	16.6 ± 14.5	73.3 ± 24.2	60.1 ± 32.0	7.9 ± 14.6	33.3 ± 19.2
**p-value** ^**b**^	< 0.001*	0.142	0.267	0.329	0.214	0.291	0.564	0.146
**Comorbidities**								
Yes	35.2 ± 17.4	63.8 ± 36.2	21.4 ± 19.4	17.6 ± 16.4	66.9 ± 24.1	54.3 ± 35.9	7.2 ± 15.1	39.9 ± 23.2
No	35.0 ± 23.2	53.4 ± 34.2	27.4 ± 24.7	13.4 ± 11.9	71.4 ± 23.8	51.7 ± 35.9	7.7 ± 15.5	56.1 ± 35.2
**p-value** ^**b**^	0.927	0.025*	0.026*	0.013*	0.120	0.540	0.717	0.097
**Monthly household Income (in Rupees)**								
≤ 25,000	34.0 ± 15.7	72.2 ± 28.4	19.0 ± 17.8	11.6 ± 11.4	66.4 ± 22.3	65.1 ± 36.8	11.8 ± 20.6	49.9 ± 33.3
> 25,000	35.3 ± 21.7	55.0 ± 36.2	25.9 ± 23.5	15.9 ± 14.5	70.0 ± 24.3	50.5 ± 35.2	6.6 ± 14.0	47.6 ± 30.5
**p-value** ^**b**^	0.706	0.004*	0.060	0.065	0.360	0.011*	0.037*	0.885

Note: ^a^One Way Anova; ^b^ T-test; *significant at 5% level of significance

#### EORTC-QLQ-C30

**Fig. 1:**
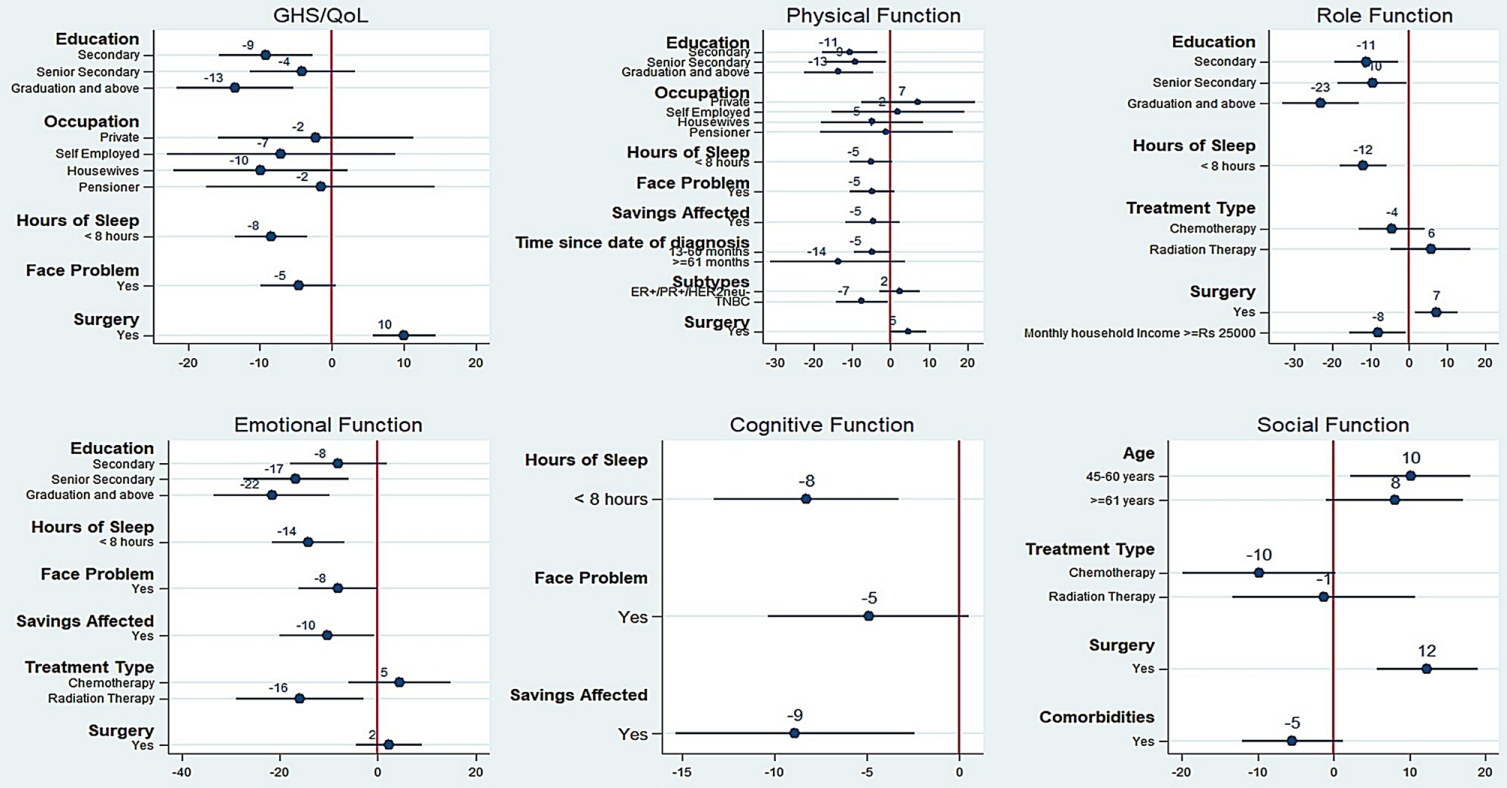
Forest plot representing regression coefficients and 95% confidence interval for EORTC-QLQ-C30 functional scores among breast cancer patients

The linear regression analysis reported the predictors associated with EORTC-QLQ-C30. Those patients who slept 8 h or more had higher GHS/QoL (β = 8.43, *p* = 0.001), role function (β = 11.93, *p* < 0.001), emotional function (β = 14.15, *p* < 0.001) and cognitive function (β = 8.26, *p* = 0.001) as compared to patients who sleep less than 8 h. Patients who had radiation therapy had lower emotional function (β =-15.90, *p* = 0.016) as compared to those who had chemotherapy (β = 4.50, *p* = 0.394). In contrast, social function was lower among the patients who underwent chemotherapy (β =-9.78, *p* = 0.057) as compared to those who underwent radiation therapy (β = -1.31, *p* = 0.829) (Fig. 1).

Those patients who slept less than 8 h faced more pain (β = 15.50, *p* < 0.001), dyspnoea (β = 8.09, *p* = 0.012), insomnia (β = 24.53, *p* < 0.001), nausea and vomiting (β = 8.45, *p* = 0.021). TNBC patients (β = 19.65, *p* = 0.05) had more insomnia as compared to ER+/PR+/HER2neu- patients. Patients who underwent radiation therapy had significantly more pain (β = 28.0, *p* < 0.001), insomnia (β = 34.16, *p* = 0.001) and diarrhoea (β = 13.36, *p* = 0.040) (Supplementary Table 1).

#### EORTC-QLQ-BR23

Patients who completed secondary education and more had fewer systemic therapy side-effects (β=-7.89, *p* = 0.033) and were less upset by hair loss (β=-14.59, *p* = 0.039) as compared to those who completed primary education. Patients who slept less than 8 h faced more systemic therapy side-effects (β = 8.30, *p* = 0.003) whereas those who slept 8 h or more had more body image (β=-9.86, *p* = 0.002) and future perspective (β=-16.77, *p* = 0.001) issues. Also, patients who underwent chemotherapy had more systemic therapy side effects, upset by hair loss whereas those who underwent radiation therapy had more arm symptoms. Similarly, the patients who underwent surgery had more arm symptoms (β = 11.86, *p* < 0.001) and those who had comorbidities had more breast symptoms (β = 5.19, *p* = 0.003) as compared to those who didn’t (Supplementary Table 2).

## Discussion

The study aimed to assess the QoL and identify its influencing factors among breast cancer patients undergoing treatment in one of the tertiary care hospitals in Pune, Maharashtra, India. The main findings in this study were:

- EORTC QLQ-30 and BR23 functioning and symptoms scales showed fatigue, pain, insomnia, financial difficulties, physical, role, emotional, social and cognitive functioning were the most affected by EORTC QLQ scales among the breast cancer patients.
- Factors associated with GHS/QoL of breast cancer patients were education, occupation, hours of sleep, financial problems, and surgery.

The mean GHS/QoL score of our study was 54.5 which was smaller than the EORTC QLQ-C30 reference value (GHS/QoL mean = 61.8, SD = 24.6) [26]. The studies conducted in China and central rural India by Chen et al. and Gangane et al. corroborate our findings, with GHS/QoL scores of 53.8 and 59.3, respectively [27, 28]. However, studies conducted in Malaysia, Ethiopia, and Morrocco conducted among breast cancer patients on QoL study reported higher scores of GHS/QoL [29–31]. In these studies, the values were 69.2 [29], 65.6 [30] and 68.5 [31]. The difference may be related to the fact that patients undergoing chemotherapy may experience various side effects that adversely affect their GHS/QoL. Also, this disparity suggests a clinically meaningful difference, as Osoba et al. (1998) indicate that a 5–10 point variation in EORTC QLQ-C30 scores can be considered significant [32]. Also, the lower GHS score in our cohort may be associated with contextual challenges such as limited access to healthcare services, socioeconomic constraints, and higher symptom burdens, including fatigue and pain, which were prevalent among our patients. Understanding these differences underscores the need for targeted interventions to address the specific needs of breast cancer patients in resource-limited settings.

Evidence from studies on Minimal Importance Difference (MID) for EORTC-QLQ-C30 suggests that a difference of 5–10 represents a small, 10–20 moderate and > 20 as large clinically meaningful difference [33]. When compared with the breast cancer cohort reporting scores around 53–59 [34, 35], the observed difference is minimal and falls below the MID threshold, suggesting comparable QoL levels, whereas studies reporting higher scores around 64–65 [36], the difference is reported to be approximately 10 points, which exceeds the MID threshold and suggests a moderate clinically meaningful difference. This states that the breast cancer patients in the present study experienced clinically relevant lower perceived overall QoL. This also indicates that the observed QoL in the present study are broadly consistent with those reported in the clinical population.

Emotional and social functioning were the most affected scales and were similar to the study conducted by Hassen et al. in Ethiopia [37]. This reduction in functioning may be related to factors reported in previous studies, such as decreased social support, treatment-related fatigue, psychological distress or caregiving responsibilities, as the majority of the participants were housewives spending most of their time at home. In the symptoms scale, severe impairment was observed in terms of fatigue, Insomnia, financial difficulties, and pain. These findings corroborate with studies conducted in Ethiopia, China, and Malaysia [29, 31, 37]. These symptoms may be associated with treatment-related side effects, which can manifest as pain, fatigue, and insomnia. Additionally, financial challenges could arise from the inability to work, indirect financial burdens such as spouses’ lost income due to caregiving responsibilities and increased medical expenses [38].

In our study, the EORTC QLQ-BR23 functional scale body image (mean = 69.5, SD = 24.0) and future perspective (mean = 52.9, SD = 35.9) were the most affected scales. These results corroborate with studies conducted by Fakir et al. in Morocco and Shin et al. in China [29, 31]. Limited social support, lack of awareness about the disease and its treatment, stigma associated with the disease, and a younger age group of patients could contribute to increased symptoms of body image and future perspective. Whereas in the symptoms scale, upset by hair loss (mean = 58.2, SD = 35.5) was the most affected scale, which was similar to a study conducted in China [29]. This may be related to the high proportion of patients undergoing chemotherapy in our sample, a treatment known to cause hair loss. Sexual functioning was also low, which may reflect treatment-related effects on body image, hormonal changes, and intimacy, highlighting an important but often under-addressed aspect of quality of life among breast cancer patients.

### Factors associated with QoL of breast cancer patients undergoing treatment

In this study, hours of sleep were a major factor associated with GHS/QoL, physical, role, emotional, and cognitive functional scales. Patients who slept less than 8 h had worse scores in the associated functional scales. Those who slept less than 8 h had more pain, nausea/vomiting, and dyspnoea as compared to those who slept 8 h or more. These findings also substantiate with findings of the study conducted by Fortner et al. [39]. However, the study used the SF-36 tool to assess the quality of life among breast cancer. This asserts that sleeping 8 h or more per day exhibited significantly higher physical functioning and global health status, supporting the model’s assertion that adequate rest is a fundamental component of physical well-being [40]. In our study, patients who underwent chemotherapy had worse scores of roles and social function whereas those who underwent radiation therapy had worse emotional function. Also, patients who had comorbidities had low social functioning and were similar to the study conducted in Ethiopia [30]. Multiple studies have also reported association between comorbidities and lower QoL [41–43]. Patients who underwent surgery had better GHS/QoL, physical, role, emotional, and social scores. Among these, patients who underwent BCS had better physical, role, emotional and social scores. These findings corroborate with the study conducted by Gupta et al. in India [13]. These findings align with Wilson and Cleary’s conceptual model of health-related QoL, which links biological and physiological factors with symptom status, functional status, and psychological well-being [44]. The improvement in social functioning among BCS compared to mastectomy patients further corroborates the World Health Organization Quality of Life (WHOQoL) model’s emphasis on social relationships, as appearance-related concerns and emotional comfort can shape interpersonal interactions and societal engagement [45, 46].

Education was significantly associated with GHS/QoL, physical, role, and emotional functioning. Patients with higher education had worse scores of GHS/QoL, along with other associated functions. On the contrary, education was not associated with GHS/QoL study in the study conducted by Getu et al. in Ethiopia [30]. This discrepancy between the studies could be due to the differences in the sample of participants in our study. In our study, the observed association does not imply reduce awareness among patients with higher education, however, may reflect differences in perceived need of care, self-management and alternative source of information during ongoing treatment. Overall, the relationship between education level and health seeking behaviour is complex, hence further research is essential to explore the contextual and psychosocial factors influencing health seeking behaviour among educated and uneducated patients. Also, the present study was not designed to examine the mechanisms underlying this relationship. The findings should be considered hypothesis-generating and warrant further investigation with a large sample size incorporating detailed psychological and behavioural assessments.

Our study highlights that pain, fatigue, insomnia, and financial difficulties significantly affect breast cancer treatment. Hence, these findings suggest the importance of conducting multicentric and longitudinal studies to better understand the QoL among breast cancer patients undergoing treatment and assess patient-reported outcome measures by incorporating clinical, psychological, financial and social factors. Furthermore, routine monitoring of QoL with standardised tools, along with patient education on self-care, can help identify and address challenges early. Also, screening for sleep disturbance and a multidisciplinary care approach will enhance symptom management; further research is needed [47]. 

### Strengths and limitations

This study, to the best of our knowledge, is the first to focus on identifying predictors associated with the quality of life of breast cancer patients undergoing treatment in India. Additionally, it is one of the few studies to consider hours of sleep as a factor affecting these patients. However, several limitations should be considered while interpreting the findings. Our study’s cross-sectional design, small sample size, and hospital-based nature limit the generalizability of our findings. Furthermore, the cross-sectional design restricts our ability to assess the quality of life of breast cancer patients across different phases of their disease and treatment. The design also limits its ability to establish temporal relationships between the identified factors and QoL outcomes. Also, all participants reported formal education, reflecting high female literacy; this may limit the generalizability of findings to a lower literacy population where awareness, access to care and health-seeking behaviour may differ. Information on the stage of the disease was not consistently available in the medical records at the time of data collection, and therefore, it could not be reliably included in the statistical analysis. The disease stage may affect the treatment intensity and overall QoL; the observed association between sociodemographic or treatment-related factors and QoL should be interpreted with caution, as potential variation could not be assessed in this study. The stage-specific data limit our ability to assess variation in QoL across different stages of disease; further studies should incorporate disease staging to enable more nuanced interpretation of QoL outcomes.

Formal effect size measures were not calculated, which may limit the interpretation of the magnitude of some observed associations. Also, another methodological limitation of our study is the variable selection strategy used for regression analysis. Some clinically relevant variables and confounders were not included in the regression model because the variables were based primarily on the statistical significance in the bivariate analysis and this approached helped reducing model complexity in studies with relatively small sample size. This process may lead to exclusion of clinically or theoretically important factors that did not reach statistical significance. Hence, the association observed in the regression analysis needs to be interpreted with caution. This observation points towards the need for a large sample size and more comprehensive clinical data to be considered in a multivariable model.

Another limitation of the study is the grouping of income into broad categories, which may not capture the income variability, socioeconomic status and the financial burden of the patient’s undergoing treatment. Also, self-reporting of income may further affect the accuracy of socioeconomic classification. These could influence the observed association between income and QoL. Hence, future studies may be considered by incorporating detailed measures of socioeconomic status (such as income, composite socioeconomic indices, household assets etc.).

## Conclusion

Our study found that various aspects of QoL, including fatigue, pain, insomnia, and financial difficulties, were significantly impacted in breast cancer patients undergoing treatment. Factors such as education, occupation, hours of sleep, financial issues, and type of surgery were linked to the patients’ QoL. Therefore, these patients require additional attention and support services, including effective pain management and interventions to enhance sleep quality, to further enhance their QoL. The study highlights the multidimensional nature of QoL among breast cancer patients undergoing treatment and underscores the importance of both clinical and sociodemographic factors when assessing patient-reported outcome measures (PROMs) in the oncology setting. Hence, incorporating routine QoL screening will help to early identify poor outcomes, enabling targeted interventions for those patients. Additionally, multicentre and longitudinal studies incorporating detailed clinical, psychological and socioeconomic variables are needed to better understand the determinants of QoL across different stages of breast cancer and its treatment.

## Electronic supplementary material

Below is the link to the electronic supplementary material.

Supplementary Material 1

## Data Availability

The datasets generated and analysed during the study are available from the authors upon reasonable request.
