# Supplementary material for: Health-related quality of life (HRQoL) of women with breast cancer undergoing treatment in a tertiary care centre in India
Source: J Patient Rep Outcomes. 2026 Apr 21;10:97. doi: 10.1186/s41687-026-01071-8 (PMC13243154; doi:10.1186/s41687-026-01071-8)
Supplement: Supplementary file 1 — Supplementary Material 1 [file 41687_2026_1071_MOESM1_ESM.docx]

**Supplementary Table-1: Linear Regression model with parameter estimates for EORTC-QLQ-C30 symptoms scores among breast cancer patients**

| **Demographic and clinical characteristics** | **Fatigue** | | **Nausea and vomiting** | | **Pain** | | **Dyspnoea** | | **Insomnia** | | **Appetite loss** | | **Constipation** | | **Diarrhoea** | | **Financial difficulties** | |
| --- | --- | --- | --- | --- | --- | --- | --- | --- | --- | --- | --- | --- | --- | --- | --- | --- | --- | --- |
|  | B (95% Confidence Interval) R2 = 0.08 | **p-value** | B (95% Confidence Interval) R2 = 0.08 | **p-value** | B (95% Confidence Interval) R2 = 0.22 | **p-value** | B (95% Confidence Interval) R2 = 0.11 | **p-value** | B (95% Confidence Interval) R2 = 0.23 | **p-value** | B (95% Confidence Interval) R2 = 0.16 | **p-value** | B (95% Confidence Interval) R2 = 0.15 | **p-value** | B (95% Confidence Interval) R2 = 0.09 | **p-value** | B (95% Confidence Interval) R2 = 0.16 | **p-value** |
| **Age (In Years)** | | | | | | | | | | | | | | | | | | |
| <45 years |  |  |  |  |  |  |  |  |  |  |  |  |  |  | REF |  | REF |  |
| 45-60 years |  |  |  |  |  |  |  |  |  |  |  |  |  |  | -2.28 (-10.27, 5.71) | 0.575 | -7.75 (-16.92,1.41) | 0.097 |
| ≥ 61 years |  |  |  |  |  |  |  |  |  |  |  |  |  |  | 4.74 (-3.99, 13.49) | 0.286 | -10.36 (-20.43, -0.30) | **0.043** |
| **Education** | | | | | | | | | | | | | | | | | | |
| Primary |  |  | REF |  | REF |  |  |  | REF |  |  |  | REF |  | REF |  | REF |  |
| Secondary |  |  | 15.94 (6.57, 25.32) | **0.001** | 6.34 (-3.83, 16.53) | 0.221 |  |  | 1.19 (-13.46, 15.85) | 0.873 |  |  | -5.23 (-15.82, 5.35) | 0.331 | 2.49 (-7.84, 12.84) | 0.635 | 8.68 (-2.65, 20.03) | 0.133 |
| Senior Secondary |  |  | 14.37 (4.03, 24.70) | **0.007** | 6.28 (-5.04, 17.62) | 0.229 |  |  | 1.62 (-14.39, 17.63) | 0.842 |  |  | 10.58 (-1.07, 22.24) | 0.075 | 6.67 (-4.27, 17.62) | 0.231 | 13.28 (1.29, 25.28) | **0.03** |
| Graduation and above |  |  | 17.91 (6.43, 29.38) | **0.002** | 18.34 (5.77, 30.91) | **0.004** |  |  | 6.31 (-11.49, 24.12) | 0.486 |  |  | -7.49 (-20.49, 5.49) | 0.257 | 18.76 (6.50, 31.01) | **0.003** | 14.84 (1.35, 28.32) | **0.031** |
| **Occupation** | | | | | | | | | | | | | | | | | | |
| Government |  |  |  |  | REF |  | REF |  | REF |  | REF |  | REF |  |  |  |  |  |
| Private |  |  |  |  | 16.52 (-4.50, 37.55) | 0.123 | -5.97 (-22.97,11.02) | 0.49 | 15.69 (-14.27, 45.66) | 0.304 | 6.88 (-16.65, 30.42) | 0.565 | 4.98 (-16.90, 26.86) | 0.654 |  |  |  |  |
| Self Employed |  |  |  |  | 12.48 (-11.94, 36.91) | 0.315 | -5.65 (-25.55, 14.24) | 0.576 | 9.66 (-24.92, 44.24) | 0.583 | 30.25 (3.08, 57.41) | **0.029** | 23.17 (-2.30, 48.65) | 0.074 |  |  |  |  |
| Housewives |  |  |  |  | 21.04 (2.29, 39.79) | **0.028** | 6.05 (-9.03, 21.15) | 0.43 | 27.81 (1.15, 54.46) | **0.041** | 35.10 (14.37, 55.82) | **0.001** | 27.98 (8.47, 47.49) | **0.005** |  |  |  |  |
| Pensioner |  |  |  |  | 6.35 (-18.72, 31.42) | 0.618 | -1.89 (-21.08, 18.02) | 0.852 | 17.58 (-17.92, 53.09) | 0.33 | 23.90 (-3.83, 51.65) | 0.091 | 20.78 (-5.16, 46.74) | 0.116 |  |  |  |  |
| **Sleep per day (in hours)** | | | | | | | | | | | | | | | | | | |
| ≥8 hours | REF |  | REF |  | REF |  | REF |  | REF |  |  |  |  |  |  |  |  |  |
| < 8 hours | 6.40 (-1.52,14.33) | 0.223 | 8.45 (1.26,15.64) | **0.021** | 15.50 (7.69, 23.31) | **0.000** | 8.09 (1.79, 14.39) | **0.012** | 24.53 (13.43, 35.63) | **0.000** |  |  |  |  |  |  |  |  |
| **Consumed traditional medicine** | | | | | | | | | | | | | | | | | | |
| No | REF |  |  |  |  |  |  |  |  |  |  |  |  |  | REF |  | REF |  |
| Yes | 10.03 (2.09, 17.97) | **0.013** |  |  |  |  |  |  |  |  |  |  |  |  | -5.60 (-13.10, 1.90) | 0.143 | 2.84 (-6.06, 11.03) | 0.568 |
| **Savings Affected** | | | | | | | | | | | | | | | | | | |
| No | REF |  |  |  |  |  |  |  |  |  |  |  |  |  |  |  | REF |  |
| Yes | 8.74 (-1.48, 18.97) | 0.093 |  |  |  |  |  |  |  |  |  |  |  |  |  |  | 18.13 (7.25, 29.00) | **0.001** |
| **Faced Financial problems** | | | | | | | | | | | | | | | | | | |
| No | REF |  |  |  |  |  | REF |  | REF |  |  |  |  |  | REF |  | REF |  |
| Yes | 7.33 (-1.26, 15.93) | 0.094 |  |  |  |  | 6.25 (-0.35, 12.86) | 0.064 | 3.69 (-8.07, 15.47) | 0.62 |  |  |  |  | 7.98 (0.08,15.87) | **0.048** | 13.36 (4.31, 22.42) | **0.004** |
| **Subtypes** | | | | | | | | | | | | | | | | | | |
| ER+/PR+/HER2neu+ |  |  |  |  |  |  |  |  | REF |  | REF |  |  |  | REF |  | REF |  |
| ER+/PR+/HER2neu- |  |  |  |  |  |  |  |  | 16.30 (5.52, 27.09) | **0.003** | -1.78 (-9.99, 6.43) | 0.67 |  |  | 5.37 (-1.92, 12.66) | 0.148 | -8.11 (-16.42, 0.19) | 0.056 |
| TNBC |  |  |  |  |  |  |  |  | 19.65 (5.85, 33.46) | **0.005** | 9.77 ( -1.03, 20.58) | 0.076 |  |  | -2.55 (11.91, 6.81) | 0.592 | -10.77 (-21.41, -0.14) | 0.047 |
| **Treatment type** | | | | | | | | | | | | | | | | | | |
| Endocrine Therapy |  |  |  |  | REF |  |  |  | REF |  | REF |  | REF |  | REF |  | REF |  |
| Chemotherapy |  |  |  |  | 2.56 (-8.42, 13.56) | 0.646 |  |  | 7.53 (-8.29, 23.35) | 0.349 | -0.32 (-12.67, 12.01) | 0.958 | 9.11 (-2.29, 20.52) | 0.117 | 4.12 (-6.37, 14.62) | 0.440 | -9.64 (-21.55,2.27) | 0.112 |
| Radiation Therapy |  |  |  |  | 28.00 (14.44, 41.57) | **0.000** |  |  | 34.16 (14.91, 53.41) | **0.001** | -16.51 (-30.96, -2.07) | **0.025** | -8.85 (-22.67, 4.96) | 0.208 | 13.36 (0.62, 26.11) | **0.040** | -18.77 (-33.39, -4.16) | **0.012** |
| **Surgery** | | | | | | | | | | | | | | | | | | |
| No |  |  | REF |  | REF |  |  |  |  |  | REF |  |  |  |  |  | REF |  |
| Yes |  |  | -8.46 (-14.74, -2.17) | **0.009** | 2.95 (-3.97, 9.87) | 0.402 |  |  |  |  | -9.23 (-16.89, -1.57) | **0.018** |  |  |  |  | -4.32 (-12.12, 3.47) | 0.276 |
| **Comorbidities** | | | | | | | | | | | | | | | | | | |
| No |  |  |  |  | REF |  |  |  | REF |  |  |  |  |  |  |  |  |  |
| Yes |  |  |  |  | -0.40 (-7.19, 6.39) | 0.907 |  |  | -4.43 (-14.18, 5.32) | 0.372 |  |  |  |  |  |  |  |  |
| **Monthly household Income (in Rupees)** | | | | | | | | | | | | | | | | | | |
| ≤25000 |  |  |  |  |  |  | REF |  |  |  |  |  |  |  | REF |  |  |  |
| >25000 |  |  |  |  |  |  | 10.59 (3.33, 17.85) | **0.004** |  |  |  |  |  |  | 7.27 (-1.68, 16.24) | 0.111 |  |  |

**Supplementary Table-2: Linear Regression model with parameter estimates for EORTC-QLQ-BR23 symptoms and functional scores among breast cancer patients**

| **Demographic and Clinical Characteristics** | **Systemic therapy Side Effects** | | **Upset by hair loss** | | **Arm Symptoms** | | **Breast Symptoms** | | **Body Image** | | **Future Perspective** | | **Sexual Functioning** | | **Sexual Enjoyment** | |
| --- | --- | --- | --- | --- | --- | --- | --- | --- | --- | --- | --- | --- | --- | --- | --- | --- |
|  | B (95% Confidence Interval) R2=0.19 | **p-value** | B (95% Confidence Interval) R2=0.11 | **p-value** | B (95% Confidence Interval) R2=0.24 | **p-value** | B (95% Confidence Interval) R2=0.14 | **p-value** | B (95% Confidence Interval) R2=0.08 | **p-value** | B (95% Confidence Interval) R2 =0.12 | **p-value** | B (95% Confidence Interval) R2=0.12 | **p-value** | B (95% Confidence Interval) R2=0.10 | **p-value** |
| **Age (In Years)** | | | | | | | | | | | | | | | | |
| <45 years | REF |  |  |  |  |  | REF |  |  |  |  |  |  |  |  |  |
| 45-60 years | -4.16 (-10.0,1.67) | 0.161 |  |  |  |  | -0.30 (-4.41, 3.81) | 0.885 |  |  |  |  |  |  |  |  |
| ≥ 61 years | -5.53 (-11.93, 0.87) | 0.09 |  | 0.0 |  |  | -3.84 (-8.50, 0.81) | 0.106 |  |  |  |  |  |  |  |  |
| **Education** | | | | | | | | | | | | | | | | |
| Primary | REF |  | REF |  | REF |  |  |  |  |  | REF |  |  |  |  |  |
| Secondary | -7.89 (-15.13, -0.64) | **0.033** | -14.59 (-28.44, -0.74) | **0.039** | 4.58 (-3.05, 12.21) | 0.238 |  |  |  |  | 0.59 (-12.18, 13.36) | 0.928 |  |  |  |  |
| Senior Secondary | 0.51 (-7.23, 8.26) | 0.896 | -9.18 (-23.84, 5.48) | 0.219 | 5.08 (-2.93, 13.11) | 0.213 |  |  |  |  | 4.66 (-9.43, 18.77) | 0.515 |  |  |  |  |
| Graduation and above | -8.08 (-16.73, 0.57) | 0.067 | -20.84 (-37.75, -3.94) | **0.016** | 4.16 (-4.90, 13.23) | 0.367 |  |  |  |  | -13.01 (-28.80, 2.76) | 0.106 |  |  |  |  |
| **Marital Status** | | | | | | | | | | | | | | | | |
| Single |  |  |  |  |  |  | REF |  |  |  |  |  |  |  |  |  |
| Married |  |  |  |  |  |  | 6.63 (-2.49, 15.76) | 0.203 |  |  |  |  |  |  |  |  |
| Divorced |  |  |  |  |  |  | 0.09 (-10.72, 10.91) | **0.02** |  |  |  |  |  |  |  |  |
| Widowed |  |  |  |  |  |  | -0.67 (-11.98, 10.63) | 0.907 |  |  |  |  |  |  |  |  |
| **Occupation** | | | | | | | | | | | | | | | | |
| Government |  |  |  |  |  |  | REF |  |  |  |  |  |  |  |  |  |
| Private |  |  |  |  |  |  | 18.95 (8.67, 29.23) | **0.000** |  |  |  |  |  |  |  |  |
| Self Employed |  |  |  |  |  |  | 12.13 (0.15, 24.10) | **0.047** |  |  |  |  |  |  |  |  |
| Housewives |  |  |  |  |  |  | 11.82 (2.73, 20.90) | **0.011** |  |  |  |  |  |  |  |  |
| Pensioner |  |  |  |  |  |  | 3.59 (-8.74, 15.93) | 0.567 |  |  |  |  |  |  |  |  |
| **Sleep per day (in hours)** | | | | | | | | | | | | | | | | |
| ≥8 hours | REF |  |  |  |  |  |  |  | REF |  | REF |  |  |  |  |  |
| <8 hours | 8.30 (2.94, 13.66) | **0.003** |  |  |  |  |  |  | -9.86 (-16.19, -3.52) | **0.002** | -16.77 ( -26.29,-7.25) | **0.001** |  |  |  |  |
| **Time since date of diagnosis** | | | | | | | | | | | | | | | | |
| ≤12 months |  |  |  |  |  |  |  |  |  |  |  |  | REF |  |  |  |
| 13-60 months |  |  |  |  |  |  |  |  |  |  |  |  | 7.78 (4.26, 11.3) | **0.000** |  |  |
| ≥61 months |  |  |  |  |  |  |  |  |  |  |  |  | 2.47 (-10.66, 15.61) | 0.633 |  |  |
| **Savings Affected** | | | | | | | | | | | | | | | | |
| No |  |  |  |  |  |  |  |  |  |  |  |  |  |  | REF |  |
| Yes |  |  |  |  |  |  |  |  |  |  |  |  |  |  | -29.23 (-57.51, -0.96) | **0.043** |
| **Subtypes** | | | | | | | | | | | | | | | | |
| ER+/PR+/HER2neu+ | REF |  |  |  | REF |  | REF |  | REF |  |  |  | REF |  |  |  |
| ER+/PR+/HER2neu- | 0.26 (-5.01,5.52) | 0.924 |  |  | 5.46 (-0.14, 11.08) | 0.056 | -1.68 (-5.23, 1.86) | 0.352 | 2.51 (-3.70, 8.74) | 0.426 |  |  | 5.03 (1.21, 8.84) | **0.010** |  |  |
| TNBC | 5.99 (-0.77, 12.75) | 0.082 |  |  | 4.38 (-2.78, 11.55) | 0.23 | 1.07 (-3.64, 5.79) | 0.654 | -4.68 (-12.82, 3.44) | 0.257 |  |  | 2.34 (-2.73, 7.43) | 0.364 |  |  |
| **Treatment type** | | | | | | | | | | | | | | | | |
| Endocrine Therapy | REF |  | REF |  | REF |  |  |  | REF |  |  |  |  |  |  |  |
| Chemotherapy | 10.80 (3.21, 18.39) | **0.005** | 18.83 (3.60, 34.06) | **0.016** | 2.35 (-5.66, 10.37) | 0.564 |  |  | -8.01 (-17.12, 1.09) | 0.084 |  |  |  |  |  |  |
| Radiation Therapy | 1.19 (-7.98, 10.37) | 0.798 | 14.21 (-5.53, 33.96) | 0.157 | 18.21 (8.45, 27.97) | **0.000** |  |  | 1.29 (-9.28, 11.87) | 0.81 |  |  |  |  |  |  |
| **Surgery** | | | | | | | | | | | | | | | | |
| No | REF |  | REF |  | REF |  |  |  | REF |  |  |  |  |  |  |  |
| Yes | -3.83 (-8.81, 1.15) | 0.131 | -8.02 (-17.21, 1.17) | 0.087 | 11.86 (6.72, 17.00) | **0.000** |  |  | 2.13 (-3.70, 7.96) | 0.473 |  |  |  |  |  |  |
| **Comorbidities** | | | | | | | | | | | | | | | | |
| No |  |  | REF |  | REF |  | REF |  |  |  |  |  |  |  |  |  |
| Yes |  |  | 5.94 (-3.33, 15.22) | 0.208 | -0.90 (-5.92, 4.12) | 0.724 | 5.19 (1.81, 8.56) | **0.003** |  |  |  |  |  |  |  |  |
| **Monthly household Income (in Rupees)** | | | | | | | | | | | | | | | | |
| ≤25000 |  |  | REF |  |  |  |  |  |  |  |  |  | REF |  |  |  |
| >25000 |  |  | -11.17 (-23.58, 1.24) | 0.078 |  |  |  |  |  |  |  |  | -5.09 (-9.80, 0.38) | **0.034** |  |  |
